# Connecticut State Dental Association

**Published:** 1865-01

**Authors:** 


Connecticut State Dental Association.—A convention of the dentists of Connecticut, held at Hartford, October 29th, 1864, resulted in the formation of a State Society under the above name, with the following list of officers:

*President*, Dr. A. Hill, of Norwalk. *Vice President*, Dr. W. W. Sheffield, of New London. *Recording Secretary*, Dr. James McManus, of Hartford. *Corresponding Secretary*, Dr. Leroy D. Pelton, of Hartford. *Treasurer*, Dr. E. E. Crofoot, of Hartford. *Librarian*, Dr. C. P. Graham, of Middletown. *Executive Committee*, Drs. Mallet, Metcalf, and Stevens, of New Haven.

